# Efficacy and safety of aspirin in patients with peripheral vascular disease: An updated systematic review and meta-analysis of randomized controlled trials

**DOI:** 10.1371/journal.pone.0175283

**Published:** 2017-04-12

**Authors:** Ahmed N. Mahmoud, Akram Y. Elgendy, Cecil Rambarat, Dhruv Mahtta, Islam Y. Elgendy, Anthony A. Bavry

**Affiliations:** 1 Department of Medicine, University of Florida, Gainesville, FL, United States of America; 2 North Florida/South Georgia Veterans Health System, Gainesville, FL, United States of America; Hvidovre Hospital, DENMARK

## Abstract

**Background:**

Although considered a cornerstone therapy, the efficacy and safety of aspirin for prevention of ischemic events in patients with peripheral vascular disease (PVD) remains uncertain. Thus, we aimed to evaluate aspirin use in both symptomatic and asymptomatic patients with PVD.

**Methods:**

An electronic search of databases was conducted from inception until January 2017 for all randomized trials comparing aspirin with either placebo or control (no aspirin) in patients with PVD. The primary efficacy outcome was all-cause mortality, and the primary safety outcome was major bleeding. Other outcomes of interest were major adverse cardiac and cerebrovascular events (MACCE), myocardial infarction (MI), stroke and intracranial hemorrhage. Random-effects summary risk ratios (RR) were calculated using Der-Simonian and Liard model. The quality of evidence was assessed by GRADE tool and Cochrane risk of bias assessment tool.

**Results:**

A total of 6,560 patients from 11 trials were included. Only two trials were considered to have low risk of bias. Compared with control, aspirin was associated with similar incidence of all-cause mortality (RR = 0.93, 95% confidence interval [CI] 0.8–1.1), MACCE (RR = 1.0, 95% CI 0.83–1.20), MI (RR = 0.91, 95% CI 0.67–1.23) and stroke (RR = 0.72, 95% CI 0.43–1.22), major bleeding (RR = 1.59, 95% CI 0.96–2.62) and intracranial hemorrhage (RR = 1.38, 95% CI 0.59–3.21).

**Conclusions:**

Aspirin use in PVD might not be associated with improved cardiovascular outcomes or worse bleeding outcomes. Larger randomized trials assessing the efficacy and safety of aspirin in the contemporary era are mandatory to confirm the current findings. Guideline recommendations regarding the use of aspirin among patients with PVD need to be updated.

## Background

Peripheral vascular disease (PVD) remains a prevalent disease worldwide, and carries significant morbidity and mortality [1]. Anti-platelet agents, particularly aspirin, have long served as the cornerstone in therapeutic management of patients with PVD [2, 3]. The benefit of aspirin as secondary prevention therapy in patients with atherosclerosis has been clearly demonstrated in patients with prior ischemic stroke or acute myocardial infarction (MI) [4, 5]. However, benefit in patients with established atherosclerosis without evidence of an ischemic event has recently been questioned [6]. Prior meta-analyses have yielded inconsistent findings with regard to the benefit of aspirin in PVD [7–9]. One reason for the disparate results of these meta-analyses is the heterogeneity of patients enrolled in the studies as well as the inclusion of studies with other antiplatelet agents, such as cilostazol or clopidogrel, as comparators. More recently, the Aspirin for Asymptomatic Atherosclerosis (AAA) trial, one of the largest randomized control trials on this topic, also failed to show a statistically significant reduction in vascular events in asymptomatic patients with PVD, adding more to the current debate [10]. Furthermore, the Examining Use of Ticagrelor in Peripheral Artery Disease (EUCLID) trial compared ticagrelor to clopidogrel in symptomatic PVD patients and failed to show any reduction in cardiovascular events with ticagrelor, questioning the general concept of antiplatelet therapy in those patients [11]. Therefore, we aimed to conduct a comprehensive meta-analysis to evaluate the efficacy and safety of aspirin in patients with PVD.

## Methods

### Data sources and study selection

As part of a project for evaluation of aspirin efficacy and safety in patients with atherosclerosis (CDR42016041548 registered on PROSPERO international prospective register of systematic reviews, https://www.crd.york.ac.uk/PROSPERO/display_record.asp?ID=CRD42016041548), an electronic search of major scientific databases including MEDLINE (**S1 File**), Web of Science and the Cochrane Central Register of Controlled Trials (CENTRAL) was conducted from inception until January 2017 without any language restrictions. The search was limited to randomized clinical trials comparing aspirin mono-therapy to placebo or no aspirin control. The following keywords were used: “aspirin”, “prevention”, “risk”, “cardiovascular”, “coronary”, “ischemia”, “stroke”, “myocardial infarction”, “carotid”, and “peripheral”. The bibliographies of the included studies and prior meta-analyses on the same topic were also screened for trials not included by this search strategy. A stepwise approach was adopted for including the final studies. First, all records were screened by the title and abstract. Studies that were potential candidates were retrieved and evaluated by two authors to insure that it would satisfy our inclusion criteria. Lastly, only studies with a primary interest of PVD were included. The current meta-analysis was conducted in concurrence with Preferred Reporting Items for Systematic Reviews and Meta-Analyses (PRISMA) guidelines [12].

### Inclusion and exclusion criteria

**Fig 1:**
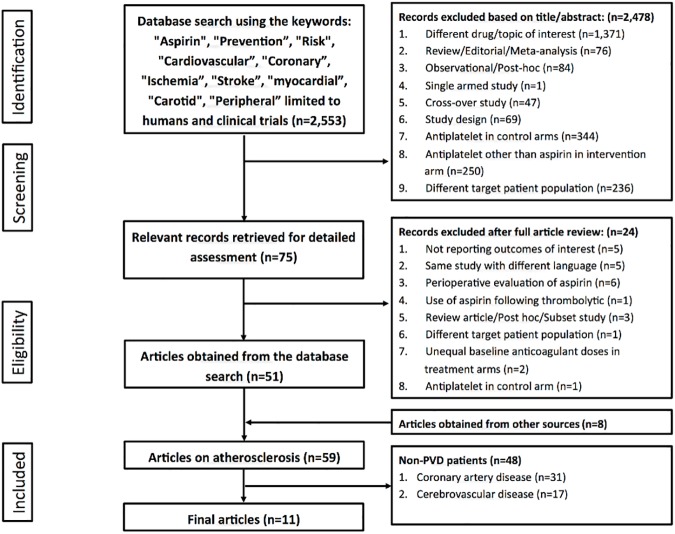
Summary of how the systematic search was conducted and eligible studies were identified (PRISMA flow diagram). PVD = peripheral vascular disease.

A study was included in the final analysis if it satisfied the following criteria: 1) a randomized controlled trial 2) comparison between aspirin therapy in one arm (i.e. without any other antiplatelet agent) versus either placebo or no aspirin control (i.e. no antiplatelet agent), 3) in adult patients (>18 years old), 4) with known history of PVD and 5) reporting cardiovascular outcomes. Exclusion criteria were: 1) patient population different than the intended focus, such as heart failure or exclusively coronary artery disease, 2) aspirin use in both arms and 3) antiplatelet agent other than aspirin in either the intervention or control arm. A more detailed listing of the study inclusion and exclusion criteria is provided in **Fig 1**.

### Data extraction

Two teams of independent authors extracted the data regarding the baseline studies characteristics, baseline patients’ demographics, studies quality data and outcomes of interest. The number of clinical events in each arm was tabulated. The first author (ANM) crosschecked all extracted data and discrepancies were resolved by consensus among authors. Outcome events were reported at the longest follow-up duration whenever possible.

### Outcomes and definitions

The outcomes of interest were categorized into two main groups; safety outcomes and efficacy outcomes. The primary efficacy outcome was all-cause mortality. All-cause mortality was chosen to represent efficacy, as it is the most robust outcome to analyze with the least degree of heterogeneity, given its universal definition. Other secondary efficacy outcomes assessed were MI, stroke and major adverse cardiac and cerebrovascular events (MACCE). The primary safety outcome was major bleeding. Intracranial hemorrhage was also assessed as a secondary safety outcome.

The definition of each outcome was reported per the individual studies (**S1 and S2 Tables**). As most of the studies were older publications, prior to the release of consensus outcomes definitions (i.e., the universal definition of MI or Bleeding Academic Research Consortium [BARC] bleeding risk score), we included events that would reflect such definitions as much as possible. MACCE outcome was reported as the composite including: mortality (cardiovascular or all-cause), MI, or stroke, whenever possible. Major bleeding compromised any bleeding that resulted in significant drop of hemoglobin (Hb) >2 gm/dL, blood transfusion, hospitalization, operation, intracranial hemorrhage or death. MI and stroke outcomes were calculated using highest number of reported events, irrespective to fatality (fatal and non-fatal).

### Quality assessment

The quality of each study included was evaluated using the Cochrane risk of bias tool [13]. This consists of seven points that test for selection, performance, detection, attrition and reporting biases. The overall level of evidence strength of each outcome was reported according to the Grades of Recommendation, Assessment, Development and Evaluation (GRADE) tool [14]. This tool reports 4 levels of quality, high, moderate, low, and very low depending on the type of studies included (e.g. observational versus randomized clinical trial). More details regarding both the Cochrane and GRADE tools are reported in the **S2 File**.

### Statistical analysis

For all descriptive analysis purposes, weighted frequencies were calculated for categorical variables and weighted means with standard deviations (SD) were calculated for continuous variables using the sample size of each trial included as the weight. Summary random effects inverse variance weighted incidences of all outcomes of interest with 95% confidence intervals (CI) were calculated using Metaprop software [15]. This method was used for calculation of pooled proportions in prior studies [16]. Summary random effects risk ratios (RR) were calculated for all outcomes of interest by Der-Simonian and Liard model [17]. Fixed effects odds ratios (OR) were calculated as a secondary confirmatory analysis for both primary efficacy and safety outcomes by Peto model [18]. The heterogeneity between studies was assessed using I^2^ statistic with values 0–30%, more than 30% to 60% and more than 60% corresponding to low, moderate and high degree of heterogeneity [19]. Publication bias was evaluated by Egger’s test with P-value of <0.05 corresponding with significant heterogeneity between studies [20].

For both the primary efficacy and safety outcomes, sensitivity analyses were conducted by excluding the largest trial [10], for low risk of bias trials, for trials explicitly reporting patients with no prior history of an ischemic event (i.e. stroke or MI), and for placebo-controlled trials only. Subgroup analysis was performed according to symptoms, that is, symptomatic or asymptomatic PVD. Random-effects meta-regression was performed for both primary outcomes according to the dose of aspirin used, percentage of diabetes mellitus, smokers, hypertension and mean age in the intervention arm. A two-sided P-value of <0.05 and CI of 95% was considered statistically significant, and all statistical analysis was performed with use of STATA software version 14 (StataCorp, College Station, Texas).

## Results

### Studies included

A total of 2,553 records were initially retrieved from the electronic database search, of which 2,478 records were excluded based on review of either the title or abstract, and 75 records were retrieved for full-text review. A final number of 11 trials reporting outcomes in patients with PDV were included in the analysis [10, 21–30] (**Fig 1**).

Three of the eleven final trials were reported in a prior meta-analysis and were not retrieved by our database search [28–30]. Two of the included trials employed factorial design and thus each trial was divided into two sub-studies for analysis purposes. The Critical Leg Ischaemia Prevention Study (CLIPS) trial randomized patients to aspirin versus placebo and aspirin plus vitamin E versus vitamin E only, while the Prevention Of Progression of Arterial Disease And Diabetes (POPADAD) trial randomized patients to aspirin versus placebo and aspirin plus antioxidant versus antioxidant only [21, 22]. Three trials had multiple comparison arms and thus only the aspirin and placebo arms were included [23, 25, 26]. Three trials were multicenter and reported outcomes exclusively in patients without prior history of an ischemic event (including prior stroke or MI) [10, 21, 22], and 4 trials included low-dose aspirin (≤ 325mg per day) [10, 21, 22, 24]. All of the trials included chronic stable PVD patients except one trial [25] that assessed the use of aspirin in setting of acute lower limb ischemia. The rest of studies’ characteristics are illustrated in **Table 1**. The primary outcome assessed by each trial is reported in **S3 Table**.

**Table 1:** Baseline study characteristics.

Study [Ref.]	Year	Single/m-multicenter	Treatment/Control	ASA dose	Duration of drug, yrs.	Follow up duration, yrs.	Patient population	Patients, n	Follow up completion
**AAA [10]**	2010	Multicenter	ASA/P	100mg QD	8.2	8.2	Stable PAD (asymptomatic)	1675/1675	99/99
**POPADAD**_**placebo**_ **[21]**	2008	Multicenter	ASA/P	100mg QD	6.7*	6.7*	Stable PAD (asymptomatic)	318/318	99/99
**POPADAD**_**antioxidant**_ **[21]**	2008	Multicenter	ASA/No aASA	100mg QD	6.7*	6.7*	Stable PAD (asymptomatic)	320/320	99/99
**CLIPS**_**placebo**_ **[22]**	2007	Multicenter	ASA/P	100mg QD	2	2	Stable PAD	91/90	63/73
**CLIPS**_**vitamins**_ **[22]**	2007	Multicenter	ASA/No ASA	100mg QD	2	2	Stable PAD	94/91	67/74
**Lassila et al. [24]**	1991	Single	ASA/No ASA	250mg QD	3M	3M	Stable PAD	72/72	NR
**Roztocil et al. [27]**	1989	Single	ASA/No ASA	400mg TID	1	1	Stable PAD	34/35	76/77
**Hess et al. [23]**	1985	Single	ASA/P	330mg TID	2	2	Stable PAD	80/80	84/86
**Green et al. [25]**	1982	Single	ASA/P	325mg TID	1	1	Acute PAD^†^	17/16	NR
**Harjola et al. [26]**	1981	Single	ASA/P	500mg TID	10D	10D	Stable PAD	92/86	100/100
**Ehresmann et al. [28]**	1977	Single	ASA/P	1500mg QD	NR	1	Stable PAD	215/213	NR
**Hess and Keil-Kur [29]**	1975	Single	ASA/P	1500mg QD	NR	2	Stable PAD	134/124	NR
**Zekert et al. [30]**	1975	Single	ASA/P	1500mg QD	14D	14D	Stable PAD	148/150	NR

All values are reported as Aspirin/Control. Follow up completion values are reported in percentage.

*Median is reported.

^†^Reperfusion was done and aspirin was administrated prior to reperfusion.

ASA: Aspirin, P: placebo, mg: milligrams, QD: once daily, TID: three times daily, Yrs: years, M: months, D: days, PAD: peripheral arterial disease, NR: not reported.

### Patients’ characteristics

A total of 6,560 patients with PVD were included in the final analysis, with 3,290 patients in the aspirin group and 3,270 patients in the placebo or no aspirin group. The mean age of the included patients was 62 (SD = 1.7) years old, 60% of the patients were women, 32% were diabetics, and 67% were smokers (current or ex-smokers). **Table 2** lists reported baseline characteristics.

**Table 2:** Baseline characteristics of the individuals enrolled in each trial.

Study [Ref.]	Age (SD) yrs.	Female	Smoking	HTN	DM	Prior MI	Prior stroke	Acute MI	Peripheral intervention
**AAA [10]**	62(6.7)/62(6.6)	71/72	65/66	NR	3/3	0/0	0/0	0/0	No
**POPADAD**_**placebo**_ **[21]**	60(10.1)/60(9.7)	58/57	65/63	NR	100/100	0/0	0/0	0/0	No
**POPADAD**_**antioxidant**_ **[21]**	61(10.0)/60(10.3)	53/57	68/68	NR	100/100	0/0	0/0	0/0	No
**CLIPS**_**placebo**_ **[22]**	64(9.4)/66(8.9)	26/18	86/84	56/61	78/73	0/0	0/0	0/0	No
**CLIPS**_**vitamins**_**[22]**	68(7.6)/67(8.3)	25/23	77/75	60/69	77/75	0/0	0/0	0/0	No
**Lassila et al. [24]**	60/60*	29/25	94/93	33/31	11/13	NR	NR	NR	Yes
**Roztocil et al. [27]**	58/59^‡^	6/11	NR	9/9	3/3	6^†^	NR	0/0	Yes
**Hess et al. [23]**	62^‖^	20/NR	NR	NR	NR	NR	NR	NR	No
**Green et al. [25]**	NR	NR	56/53	NR	NR	NR	NR	NR	Yes
**Harjola et al. [26]**	57/58^‡^	18/23	NR	NR	NR	NR	NR	NR	Yes
**Ehresmann et al. [28]**	NR	NR	NR	NR	NR	NR	NR	NR	No
**Hess and Keil-Kur [29]**	NR	NR	NR	NR	NR	NR	NR	NR	No
**Zekert et al. [30]**	NR	NR	NR	NR	NR	NR	NR	NR	No

All values are reported as Aspirin/Control. All values are reported in percentage except age as means and standard deviations. Smoking history is defined as either current smoking or past history of smoking.

Ref.: references, SD: standard deviation, Yrs.: years, HTN: hypertension, DM: diabetes mellitus, MI: myocardial infarction, TIA: transient ischemic attack, NR: not reported.

* Age range was 37–84 years and 36–80 years in both the aspirin and control arms, respectively.

^†^ A total of 4 patients with history of prior MI in the whole cohort.

^‡^ The age distribution was not reported in both Roztocil, et al. and Harjola et al.

^‖^ Mean age of the whole cohort.

### Quality assessment

At the individual study level, two trials were considered to have low risk of bias assessed with use of the Cochrane risk of bias tool [10, 21]. One trial was considered to have high risk of bias [22], and the remaining trials had unclear risk due to lack of reporting of detailed methods (**S4 Table**). At the outcomes level, the level of evidence was considered low to moderate in strength as assessed with use of the GRADE assessment tool (**S5 Table**).

### Primary efficacy outcome

**Fig 2:**
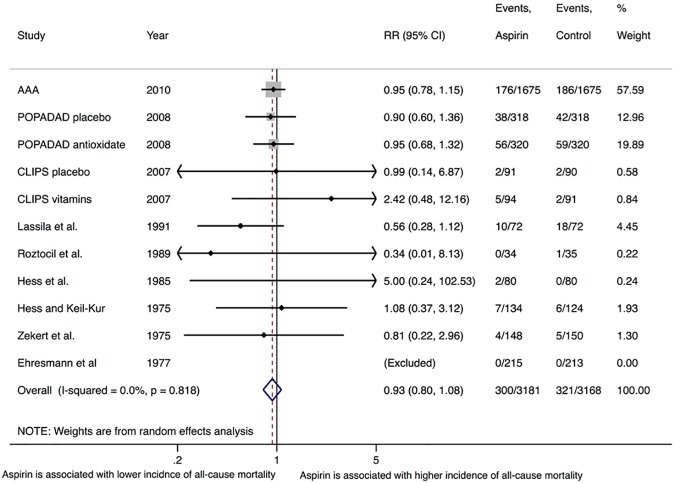
Random-effects summary plot for the primary efficacy outcome of all-cause mortality. The relative size of the data markers indicates the weight of the sample size from each study. CI = confidence interval, RR = risk ratio. P-value represents Chi-square test of heterogeneity.

Nine trials reported the outcome of all-cause mortality [10, 21–24, 27–30]. At a weighted mean follow up duration of 6.1 (SD = 2.9) years, the incidence of all-cause mortality was similar in both the aspirin and control groups, 7.7% (95% CI 4.3–11.1%) versus 8.5% (95% CI 4.6–12.5%) (RR = 0.93, 95% CI 0.8–1.1, P = 0.33, I^2^ = 0%) (**Fig 2**). The same was true by fixed effects (OR = 0.92, 95% CI 0.78–1.09, P = 0.36). Sensitivity analyses yielded similar results after exclusion of the largest trial (RR = 0.90, 95% CI 0.69–1.17, P = 0.44, I^2^ = 0%), when low risk of bias trials were evaluated individually (RR = 0.94, 95% CI 0.81–1.10, P = 0.44, I^2^ = 0%), when limited to trials explicitly reporting patients without prior history of ischemic events (RR = 0.95, 95% CI 0.81–1.11, P = 0.51, I^2^ = 0%), and when limited to placebo-controlled trials only (RR = 0.95, 95% CI 0.80–1.12, P = 0.52, I^2^ = 0%). The same was true for the subgroup according to symptoms (**S1 Fig**). Meta-regression by the dose of aspirin used, percentage of diabetes mellitus, smokers, hypertension, and mean age in the intervention arm did not show any evidence of effect modification by such variables with P-values ≥0.05. There was no evidence of publication bias by Egger’s test (P = 0.77).

### Secondary efficacy outcomes

**Fig 3:**
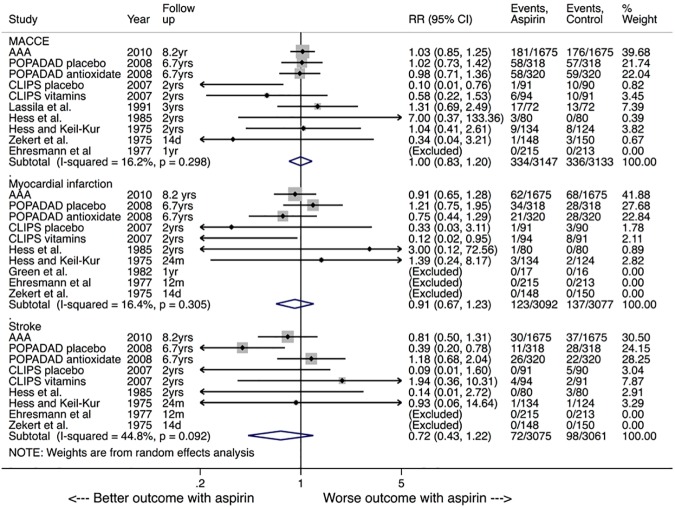
Random-effect summary plot for major adverse cardiac and cerebrovascular events (MACCE), myocardial infarction (MI) and stroke. The relative size of the data markers indicates the weight of the sample size from each study. CI = confidence interval, RR = risk ratio. P-values represent Chi-square test of heterogeneity.

Eight studies reported the incidence of MI [10, 21–23, 25, 28–30]. At a weighted mean follow up of 6.2 (SD = 2.9) years the incidence of MI was similar in both the aspirin and control groups, 3.6% (95% CI 1.63–5.5%) versus 5.5% (95% CI 3.15–7.86%), respectively; RR = 0.91, 95% CI 0.67–1.23, P = 0.54, I^2^ = 16%. Stroke was reported by 7 trials with a weighted mean follow up duration of 6.2 (SD = 2.9) years [10, 21–23, 28–30]. The incidence of stroke was 3.2% (95% CI 1.3–5.1%) in the aspirin group versus 4% (95% CI 2.0–6.0%) in the control group (RR = 0.72, 95% CI 0.43–1.22, P = 0.22, I^2^ = 45%). MACCE was reported by 9 trials at a weighted mean follow up duration of 6.1 (SD = 2.9) years [10, 21–24, 28–30]. The incidence of MACCE was similar between both groups, 9.3% (95% CI 4.8–13.9%) versus 11.6% (95% CI 7.2–16.0%), respectively, (RR = 1.0, 95% CI 0.83–1.20, P = 0.96, I^2^ = 16%) (**Fig 3**). There was no evidence of publication bias for any of the secondary efficacy outcomes (P-values of 0.51, 0.46 and 0.38 for MI, stroke and MACCE, respectively).

### Primary safety outcome

**Fig 4:**
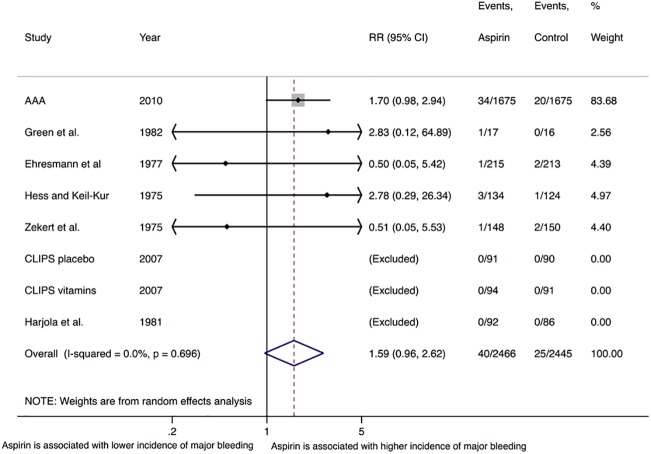
Random-effects summary plot for the primary safety outcome of major bleeding. The relative size of the data markers indicates the weight of the sample size from each study. CI = confidence interval, RR = risk ratio. P-value represents Chi-square test of heterogeneity.

The primary safety outcome of major bleeding was reported by 7 trials [22, 25, 26, 28–31]. At a weighted mean follow up duration of 5.9 (SD = 3.3) years, there was no difference incidence of major bleeding with aspirin (1.3% [95% CI 0.3–2.3]) compared with controls (1.1% [95% CI 0.7–1.6]), by either random effect (RR = 1.59, 95% CI 0.96–2.62, P = 0.07, I^2^ = 0%) (**Fig 4**) or fixed effects model (OR = 1.59, 95% CI 0.97–2.59, P = 0.06). Sensitivity analyses, excluding AAA (RR = 1.12, 95% CI 0.32–3.83, P = 0.87, I^2^ = 0%) and limited to placebo-controlled trials only (RR = 1.59, 95% CI 0.96–2.62, P = 0.07, I^2^ = 0%) yielded the same results. Other subgroup and sensitivity analyses could not be performed, as AAA was the only high quality trial, reporting outcomes in asymptomatic patients and explicitly reporting outcomes in patients without prior history of ischemic events subgroups [10]. Meta-regression analysis by the dose of aspirin used did not show any evidence of effect modification (P = 0.50); other variables were limited to conduct a meta-regression analysis. There was no evidence of publication bias by Egger’s test (P = 0.54).

### Secondary safety outcome

Only two trials reported the outcome of intracranial hemorrhage [10, 21]. At a weighted mean follow up of 7.8 (SD = 0.7) years the incidence of intracranial hemorrhage was 0.7% (95% CI 0.3–1.0%) in the aspirin group versus 0.4% (95% CI 0.2–0.7%) in the control group (RR = 1.38, 95% CI 0.59–3.21, P = 0.46, I^2^ = 0%).

## Discussion

In this meta-analysis of 11 randomized controlled trials, we compared aspirin mono-therapy to either placebo or control (no aspirin) in patients with PVD, and demonstrated lack of benefit of aspirin in these patients. The incidences of all-cause mortality, MACCE, MI, and stroke were similar between both groups across a wide range of aspirin dosages. At the same time, aspirin was not associated with an increased risk of either major bleeding or intracranial hemorrhage.

Aspirin use in PVD was recently endorsed by the American College of Cardiology (ACC) / American Heart Association (AHA) PVD guidelines in both symptomatic (Class I, level of evidence A recommendation) and asymptomatic (Class IIa, level of evidence C) patients [2]. However, these recommendations were based mainly on 3 studies: a single randomized trial [22] which had a high risk of bias by the Cochrane risk assessment tool; a meta-analysis by Berger et al [7] that showed a trend of reduction in the composite of MI, nonfatal stroke, and cardiovascular death with aspirin that did not reach statistical significance; together with older evidence from the Antithrombotic Trialists’ Collaboration (ATC) meta-analysis [4]. The ATC meta-analysis, which consisted of 42 trials including 9,717 patients, established the effectiveness of anti-platelet therapy by showing a 23% reduction in adverse cardiovascular events in patients with PVD treated with anti-platelet agents as compared to controls. However, the majority of the PVD trials included in the ATC meta-analysis consisted of anti-platelet agents other than aspirin and hence the effectiveness of aspirin was brought into question [4].

Indeed, the current meta-analysis of randomized trials has some major differences compared to the meta-analysis by Berger et al. [7]. Although the former analysis showed a trend toward benefit of aspirin in reducing the composite of MI, nonfatal stroke, and cardiovascular death, together with a significant reduction of non-fatal stroke, it did not include the largest trial to evaluate the benefit of aspirin in PVD patients [10] and excluded fatal stroke from the analysis of stroke outcome. Our analyses showed a trend toward risk reduction of stroke that did not achieve statistical significance by random-effects model, when both fatal and non-fatal stroke events were included.

Interestingly, the dose of aspirin appeared to have a minor effect on major bleeding in our analysis. Although this might seem to be counter-intuitive, given prior evidence of higher risk of gastrointestinal bleeding with increasing aspirin doses [32], this might be explained by the low number and small size of the included trials. An estimated trial with sample size of approximately 7000 patients in each arm would be required to calculate a statistically significant 50% increase in major bleeding in the aspirin receiving arm, with 80% power and 0.05 alpha. Similarly the current sample size of this meta-analysis was enough to evaluate a 20% decrease in mortality in the aspirin arm compared with controls. However, a larger sample size is required to evaluate aspirin benefits at lower rates. This mandates the conduction of a larger randomized clinical trial to further assess both the risk and benefit of aspirin in PVD patients.

It is worth mentioning that more than 90% of the current analysis weight was contributed by 3 trials explicitly reporting outcomes in PVD patients without prior history of an ischemic event with the majority of patients being asymptomatic [10, 21, 22]. This is concurrent with prior evidence from large observational studies illustrating the lack of aspirin benefit in patients with known history of coronary artery disease with no prior ischemic event (i.e. MI or stroke) [6]. With that being said, the results of the current meta-analysis should not be generalized to cover all patients with PVD, as the majority of PVD patients had asymptomatic disease with small number of patients with symptomatic or acute disease.

To fit the current findings into clinical practice, several factors should be taken into consideration including: benefit-risk balance of aspirin therapy and possible non-cardiovascular or cerebrovascular benefits of aspirin in the general population. Although the results of the current study showed a neutral effect of aspirin from a cardiovascular and major bleeding standpoint, the use of aspirin might be advocated for alternative factors, such as its anti-inflammatory and anti-tumorigenesis effects. One example is the prevention of colorectal cancer, as outlined in the 2016 United State Preventive Services Task Force recommendation statement [33]. At the same time, aspirin therapy is considerably cheaper than alternative antiplatelet and anticoagulant agents, which are sometimes used for treatment of PVD, a factor that may potentiate compliance issues in patients with low socioeconomic status. Another important aspect to consider is the possible association of increased risk of recurrent ischemic events following abrupt discontinuation of aspirin, especially in patients with recent acute ischemic events or percutaneous coronary interventions [34].

To our knowledge, the current meta-analysis of randomized trials represents the most updated analysis comparing aspirin mono-therapy to placebo or no aspirin control. Despite the large number of patients, the vigorous selection criteria and the conduction of various sensitivity and meta-regression analyses, this study is not free from limitations. Limitations of the current meta-analysis include the large weight of a single trial that might have driven some of the outcomes toward its side. We attempted to overcome such limitation by conduction of a sensitivity analysis excluding this study and showed similar results. Some of the outcomes, such as stroke had evidence of moderate heterogeneity. We used random-effects models to account for such heterogeneity. Moreover, we conducted various meta-regression and sensitivity analyses to explore the heterogeneity. Most of the included trials were older publications prior to the statin era. Another limitation was the lack of outcome definition in many of the included trials, which were conducted before the establishment of consensus outcome definitions, such as BARC for major bleeding or the universal definition of MI. We attempted to overcome such limitation by including reported outcomes that closely fit those definitions whenever feasible. Finally, the lack of patient level data hindered the assessment of aspirin efficacy and safety in certain patient subgroups such as the elderly, women, or diabetic patients.

In conclusion, aspirin use in PVD patients might not be associated with improved cardiovascular outcomes. However, most of the trials addressing the topic appear to be older trials with unclear risk of bias and outcomes definitions. Thus, larger randomized trials assessing the efficacy and safety of aspirin in the contemporary era of statins use are mandatory to confirm the current findings. The universal recommendation for aspirin therapy among patients with stable PVD needs to be re-examined.

## Supporting information

S1 Checklist PRISMA checklist. (DOC)

S1 File Medline search key used in the current analysis. (DOCX)

S2 File Detailed description of Cochrane and GRADE tools. (DOCX)

S1 Table Definition of the efficacy outcomes per each trial. (DOCX)

S2 Table Definition of the safety outcomes per each trial. (DOCX)

S3 Table Definition of the primary outcome per each trial. (DOCX)

S4 Table Cochrane risk of bias assessment tool. (DOCX)

S5 Table GRADE level of evidence assessment tool. (DOCX)

S1 Fig Subgroup random effects summary risk ratio subgroup according to symptoms. (DOCX)
