# Supplementary material for: Efficacy and safety of aspirin in patients with peripheral vascular disease: An updated systematic review and meta-analysis of randomized controlled trials
Source: PLoS One. 2017 Apr 12;12(4):e0175283. doi: 10.1371/journal.pone.0175283 (PMC5389721; doi:10.1371/journal.pone.0175283)
Supplement: S1 File — (DOCX) [file pone.0175283.s002.docx]

**S1 File. Medline search key used in the current analysis.**

("aspirin"[Mesh]) AND ("Prevention"[All fields] OR "Risk"[All fields] OR "Cardiovascular"[All fields] OR "Coronary"[All fields] OR "Ischemia"[All fields] OR "Stroke"[All fields] OR "myocardial infarction"[All fields] OR "Transient ischemic attack"[All fields] OR "Bypass"[All fields] OR "PCI"[All fields] OR "Angioplasty"[All fields] OR "Carotid"[All fields] OR "Peripheral"[All fields])) AND (Randomized Controlled Trial[ptyp] AND Humans[Mesh]).
