# Supplementary material for: Efficacy and safety of aspirin in patients with peripheral vascular disease: An updated systematic review and meta-analysis of randomized controlled trials
Source: PLoS One. 2017 Apr 12;12(4):e0175283. doi: 10.1371/journal.pone.0175283 (PMC5389721; doi:10.1371/journal.pone.0175283)
Supplement: S2 File — (DOCX) [file pone.0175283.s003.docx]

**S2 File.** **Detailed description of the Cochrane and GRADE tools.**

The Cochrane Collaboration’s tool for assessing risk of bias is the recommended tool for assessment of individual trials risk of bias by the Cochrane Collaboration. It is compromised of 7 points that assess for selection (Random sequence generation and Allocation concealment), performance (Blinding of participants and personnel), detection (Blinding of outcome assessment), attrition (Incomplete outcome data), reporting (Selective reporting) and other sources of bias. As recommended by the Cochrane handbook for systematic reviews we avoiding using scales for assessment of each study risk of bias and used the prior stated points to assess for the empirical evidence of bias, likely direction of bias and magnitude of bias. A final risk score was given to each trial (either low, high or unclear) depending on the reviewers’ assessment.^12^

The Grades of Recommendation, Assessment, Development and Evaluation (GRADE) tool specifics four levels of quality (high, moderate, low and very low) depending on the type of studies included in the assessment of each outcome, where a randomized clinical trial received the highest level of evidence, however, the quality of evidence could be downgraded depending on 5 factors: 1) limitations in the design and implementation of available studies (i.e., high likelihood of bias), 2) indirectness of evidence (indirect population, intervention, control, and outcomes), 3) unexplained heterogeneity or inconsistency of results, 4) imprecision of results (i.e. wide confidence intervals), and 5) high probability of publication bias.^13^
