# Supplementary material for: Efficacy and safety of aspirin in patients with peripheral vascular disease: An updated systematic review and meta-analysis of randomized controlled trials
Source: PLoS One. 2017 Apr 12;12(4):e0175283. doi: 10.1371/journal.pone.0175283 (PMC5389721; doi:10.1371/journal.pone.0175283)
Supplement: S1 Table — (DOCX) [file pone.0175283.s004.docx]

**S1 Table.** **Definition of the efficacy outcomes per each trial.**

| Study, reference | MACCE | CV mortality | Myocardial infarction | Stroke |
| --- | --- | --- | --- | --- |
| AAA^10^ | Composite of initial fatal or nonfatal coronary event or stroke or revascularization | Summation of fatal stroke and coronary events | Nonfatal MI | Ischemic stroke |
| POPADAD ^21^ | Death from coronary heart disease or stroke, nonfatal MI, stroke, or above ankle amputation for critical limb ischemia | Death from coronary heart disease or stroke | Nonfatal MI | All strokes (fatal/nonfatal) |
| CLIPS ^22^ | Fatal and nonfatal MI, stroke or PE | Fatal MI and stroke (PE excluded) | Any two of: ischemic chest pain >20 minutes, CK, CK-MB, LDH and AST greater than 2x upper limit of normal, new Q wave in at least 2 adjacent leads and new dominant R wave in 1 lead | Any non-hemorrhagic acute neurological vascular event with focal signs lasting >24 hours if in a new location or accompanied by new CT or MRI findings (or more than 7 days if it was the worsening of a previous deficit) |
| Lassila et al.^24^ | Death or new cardiovascular events | Death from CV event (angina pectoris, acute MI, TIA, or stroke) | NR | NR |
| Roztocil et al.^27^ | NR | Death from MI | Not defined | Not defined |
| Hess et al.^23^ | NR | NR | NR | NR |
| Green et al.^25^ | NR | NR | NR | NR |
| Harjola et al.^26^ | NR | NR | NR | NR |
| Ehresmann et al.^28^ | Composite cardiovascular end points | NR | Nonfatal MI | Nonfatal stroke |
| Hess and Keil-Kur^29^ | Composite cardiovascular end points | NR | Nonfatal MI | Nonfatal stroke |
| Zekert et al.^30^ | Composite cardiovascular end points | NR | Nonfatal MI | Nonfatal stroke |

MACCE: major adverse cardiac and cerebrovascular events, CV: cardiovascular, MI: myocardial infarction, PE: pulmonary embolism, TIA: transient ischemic attack, NR: not reported, CT: computerized tomography, MRI: magnetic resonance imaging.
