# Supplementary material for: Efficacy and safety of aspirin in patients with peripheral vascular disease: An updated systematic review and meta-analysis of randomized controlled trials
Source: PLoS One. 2017 Apr 12;12(4):e0175283. doi: 10.1371/journal.pone.0175283 (PMC5389721; doi:10.1371/journal.pone.0175283)
Supplement: S2 Table — (DOCX) [file pone.0175283.s005.docx]

**Supplemental Table 2:** Definition of the safety outcomes per each trial.

| Study, reference | Major bleeding | ICH or hemorrhagic stoke |
| --- | --- | --- |
| AAA^10^ | Admission to the hospital to control the bleeding | Hemorrhagic stroke and subarachnoid/subdural based on brain scan |
| POPADAD ^21^ | NR | Fatal hemorrhagic stroke only |
| CLIPS ^22^ | Not defined (Authors stated that all bleeding was mild/moderate) | Primary intracranial hemorrhage (intracerebral, subarachnoid or subdural) documented by CT or MRI |
| Lassila et al.^24^ | NR | NR |
| Roztocil et al.^27^ | NR | NR |
| Hess et al.^23^ | NR | NR |
| Green et al.^25^ | NR | NR |
| Harjola et al.^26^ | Not defined (No bleeding events) | NR |
| Ehresmann et al.^28^ | NR | NR |
| Hess and Keil-Kur^29^ | NR | NR |
| Zekert et al.^30^ | NR | NR |

ICH: intracranial hemorrhage, MI: myocardial infarction, PE: pulmonary embolism, NR: not reported, CT: computerized tomography, MRI: magnetic resonance imaging.
