# Supplementary material for: Efficacy and safety of aspirin in patients with peripheral vascular disease: An updated systematic review and meta-analysis of randomized controlled trials
Source: PLoS One. 2017 Apr 12;12(4):e0175283. doi: 10.1371/journal.pone.0175283 (PMC5389721; doi:10.1371/journal.pone.0175283)
Supplement: S3 Table — (DOCX) [file pone.0175283.s006.docx]

**S3 Table. Definition of the primary outcome per each trial.**

| Study, reference | Primary outcome |
| --- | --- |
| AAA^10^ | Composite of initial fatal or nonfatal coronary event or stroke or revascularization |
| POPADAD ^21^ | Death from coronary heart disease or stroke, non-fatal MI or stroke, or above ankle amputation for critical limb ischemia |
| CLIPS ^22^ | Fatal and nonfatal vascular events (MI, stroke and PE) and critical leg ischemia |
| Lassila et al.^24^ | Death or new cardiovascular events |
| Roztocil et al.^27^ | Maximum Calf blood flow |
| Hess et al.^23^ | Peripheral arterial atherosclerotic changes |
| Green et al.^25^ | Graft patency |
| Harjola et al.^26^ | Graft occlusion |
| Ehresmann et al.^28^ | Graft occlusion |
| Hess and Keil-Kur^29^ | Patency rates |
| Zekert et al.^30^ | Graft occlusion |

MI: myocardial infarction, PE: pulmonary embolism.
