# Supplementary material for: Efficacy and safety of aspirin in patients with peripheral vascular disease: An updated systematic review and meta-analysis of randomized controlled trials
Source: PLoS One. 2017 Apr 12;12(4):e0175283. doi: 10.1371/journal.pone.0175283 (PMC5389721; doi:10.1371/journal.pone.0175283)
Supplement: S4 Table — (DOCX) [file pone.0175283.s007.docx]

**S4 Table.** **Cochrane risk of bias assessment tool.**

| Study, reference | Random sequence generation | Allocation concealment | Blinding of participants and personnel | Blinding of outcome assessment | Incomplete outcome data | Selective reporting | Overall risk of bias |
| --- | --- | --- | --- | --- | --- | --- | --- |
| AAA^10^ | Permuted blocks of eight and was computer generated | Sealed envelope | Double blind | Yes | Low risk | Low risk | Low risk |
| POPADAD ^21^ | Permuted blocks of eight and was computer generated | Independent pharmacist packaged the drugs into numbered containers | Double blind | Yes | Low risk | Low risk | Low risk |
| CLIPS ^22^ | Unclear | Sealed envelope off-site by a central facility | Double blind | No | High risk | Low risk | High risk |
| Lassila et al.^24^ | Unclear | Unclear | Unclear | Unclear | Unclear | Low risk | Unclear |
| Roztocil et al.^27^ | Unclear | Unclear | Double blind | Unclear | Unclear | Unclear | Unclear |
| Hess et al.^23^ | Unclear | Unclear | Double blind | Unclear | Unclear | Unclear | Unclear |
| Green et al.^25^ | Unclear | Sealed envelope | Unclear | Unclear | Unclear | Unclear | Unclear |
| Harjola et al.^26^ | Unclear | Unclear | Unclear | Unclear | Unclear | Unclear | Unclear |
| Ehresmann et al.^28^ | Unclear | Unclear | Unclear | Unclear | Unclear | Unclear | Unclear |
| Hess and Keil-Kur^29^ | Unclear | Unclear | Unclear | Unclear | Unclear | Unclear | Unclear |
| Zekert et al.^30^ | Unclear | Unclear | Unclear | Unclear | Unclear | Unclear | Unclear |
