# Supplementary material for: Efficacy and safety of aspirin in patients with peripheral vascular disease: An updated systematic review and meta-analysis of randomized controlled trials
Source: PLoS One. 2017 Apr 12;12(4):e0175283. doi: 10.1371/journal.pone.0175283 (PMC5389721; doi:10.1371/journal.pone.0175283)
Supplement: S5 Table — (DOCX) [file pone.0175283.s008.docx]

**S5 Table. GRADE level of evidence assessment tool.**

| **Quality assessment** | | | | | | | **№ of patients** | | **Effect** | | **Quality** | **Importance** |
| --- | --- | --- | --- | --- | --- | --- | --- | --- | --- | --- | --- | --- |
| **№ of studies** | **Study design** | **Risk of bias** | **Inconsistency** | **Indirectness** | **Imprecision** | **Other considerations** | **Aspirin** | **control** | **Relative (95% CI)** | **Absolute (95% CI)** |  |  |
| All-cause mortality | | | | | | | | | | | | |
| 9 | randomized trials | serious ^a,b^ | not serious ^c^ | not serious | not serious | none ^d^ | 300/3181 (9.4%) | 321/3168 (10.1%) | **RR 0.93** (0.80 to 1.08) | **7 fewer per 1,000** (from 8 more to 20 fewer) | ⨁⨁⨁◯ MODERATE | IMPORTANT |
| Myocardial infarction | | | | | | | | | | | | |
| 8 | randomized trials | serious ^a,b^ | not serious ^e^ | not serious | not serious | none ^d^ | 123/3092 (4.0%) | 137/3077 (4.5%) | **RR 0.91** (0.67 to 1.23) ^f^ | **4 fewer per 1,000** (from 10 more to 15 fewer) ^f^ | ⨁⨁⨁◯ MODERATE | IMPORTANT |
| Stroke | | | | | | | | | | | | |
| 7 | randomized trials | serious ^a,b^ | serious ^g^ | not serious | not serious | none ^d^ | 72/3075 (2.3%) | 98/3061 (3.2%) | **RR 0.72** (0.43 to 1.22) ^f^ | **9 fewer per 1,000** (from 7 more to 18 fewer) ^f^ | ⨁⨁◯◯ LOW | IMPORTANT |
| Major bleeding | | | | | | | | | | | | |
| 7 | randomized trials | serious ^a,b^ | not serious ^h^ | not serious | not serious | none ^d^ | 40/2466 (1.6%) | 25/2445 (1.0%) | **RR 1.59** (0.96 to 2.62) ^f^ | **6 more per 1,000** (from 0 fewer to 17 more) ^f^ | ⨁⨁⨁◯ MODERATE | IMPORTANT |
| Intracranial hemorrhage | | | | | | | | | | | | |
| 2 | randomized trials | serious ^a,b^ | not serious ^i^ | not serious | serious ^j^ | none ^d^ | 13/2313 (0.6%) | 10/2313 (0.4%) | **RR 1.38** (0.59 to 3.21) ^f^ | **2 more per 1,000** (from 2 fewer to 10 more) ^f^ | ⨁⨁◯◯ LOW |  |

**CI:** Confidence interval; **RR:** Risk ratio

a. Risk of bias was assessed by the Cochrane Collaboration’s tool

b. Most of the reported outcomes lacked definition by the study authors

c. I-squared (variation in RR attributable to heterogeneity)=0%

d. No evidence of publication bias by Egger's test

e. I-squared (variation in RR attributable to heterogeneity)=16%

f. Non-weighted absolute risk reductions are calculated

g. I-squared (variation in RR attributable to heterogeneity)=45%

h. I-squared (variation in RR attributable to heterogeneity)=0%

i. I-squared (variation in RR attributable to heterogeneity)=0%

j. Confidence internal= 0.59-3.21
