# Supplementary material for: Efficacy and safety of aspirin in patients with peripheral vascular disease: An updated systematic review and meta-analysis of randomized controlled trials
Source: PLoS One. 2017 Apr 12;12(4):e0175283. doi: 10.1371/journal.pone.0175283 (PMC5389721; doi:10.1371/journal.pone.0175283)
Supplement: S1 Fig — (DOCX) [file pone.0175283.s009.docx]

**S1 Fig.** **Subgroup random effects summary risk ratio subgroup according to symptoms.**

* CLIPS trials had approximately 25% asymptomatic PVD patients.

Aspirin is associated with lower incidence of all-cause mortality

Aspirin is associated with higher incidence of all-cause mortality
